# Standardization and Validation of Brachytherapy Seeds’ Modelling Using GATE and GGEMS Monte Carlo Toolkits

**DOI:** 10.3390/cancers13215315

**Published:** 2021-10-22

**Authors:** Konstantinos P. Chatzipapas, Dimitris Plachouris, Panagiotis Papadimitroulas, Konstantinos A. Mountris, Julien Bert, Dimitris Visvikis, Dimitris Mihailidis, George C. Kagadis

**Affiliations:** 1 3DMI Research Group, Department of Medical Physics, School of Medicine, University of Patras, 26504 Rion, Greece; kwnchatz@upatras.gr (K.P.C.); dim.plachouris@gmail.com (D.P.); 2 Bioemission Technology Solutions (BIOEMTECH), 15343 Athens, Greece; panpap@bioemtech.com; 3 Aragon Institute of Engineering Research, IIS Aragon, University of Zaragoza, 50018 Zaragoza, Spain; konstantinos.mountris@gmail.com; 4 LaTIM, INSERM, UMR1101, Camille Desmoulins Av. 22, 29200 Brest, France; julien.bert@univ-brest.fr (J.B.); dimitris@univ-brest.fr (D.V.); 5 Radiation Oncology, University of Pennsylvania, Philadelphia, PA 19104, USA; dimitris@charlestonradiation.com

**Keywords:** brachytherapy, TG-43, GATE, GGEMS, Monte Carlo simulations, TG-186

## Abstract

**Simple Summary:**

This study used GATE and GGEMS simulation toolkits, to estimate dose distribution on Brachytherapy procedures. Specific guidelines were followed as defined by the American Association of Physicists in Medicine (AAPM) as well as by the European SocieTy for Radiotherapy and Oncology (ESTRO). Several types of brachytherapy seeds were modelled and simulated, namely Low-Dose-Rate (LDR), High-Dose-Rate (HDR), and Pulsed-Dose-Rate (PDR). The basic difference between GATE and GGEMS is that GGEMS incorporates GPU capabilities, which makes the use of Monte Carlo (MC) simulations more accessible in clinical routine, by minimizing the computational time to obtain a dose map. During the validation procedure of both codes with protocol data, differences as well as uncertainties were measured within the margins defined by the guidelines. The study concluded that MC simulations may be utilized in clinical practice, to optimize dose distribution in real time, as well as to evaluate therapeutic plans.

**Abstract:**

This study aims to validate GATE and GGEMS simulation toolkits for brachytherapy applications and to provide accurate models for six commercial brachytherapy seeds, which will be freely available for research purposes. The AAPM TG-43 guidelines were used for the validation of two Low Dose Rate (LDR), three High Dose Rate (HDR), and one Pulsed Dose Rate (PDR) brachytherapy seeds. Each seed was represented as a 3D model and then simulated in GATE to produce one single Phase-Space (PHSP) per seed. To test the validity of the simulations’ outcome, referenced data (provided by the TG-43) was compared with GATE results. Next, validation of the GGEMS toolkit was achieved by comparing its outcome with the GATE MC simulations, incorporating clinical data. The simulation outcomes on the radial dose function (RDF), anisotropy function (AF), and dose rate constant (DRC) for the six commercial seeds were compared with TG-43 values. The statistical uncertainty was limited to 1% for RDF, to 6% (maximum) for AF, and to 2.7% (maximum) for the DRC. GGEMS provided a good agreement with GATE when compared in different situations: (a) Homogeneous water sphere, (b) heterogeneous CT phantom, and (c) a realistic clinical case. In addition, GGEMS has the advantage of very fast simulations. For the clinical case, where TG-186 guidelines were considered, GATE required 1 h for the simulation while GGEMS needed 162 s to reach the same statistical uncertainty. This study produced accurate models and simulations of their emitted spectrum of commonly used commercial brachytherapy seeds which are freely available to the scientific community. Furthermore, GGEMS was validated as an MC GPU based tool for brachytherapy. More research is deemed necessary for the expansion of brachytherapy seed modeling.

## 1. Introduction

The estimation of radiation dose is a standard step followed at every radiation treatment procedure. The planning systems used in brachytherapy (BT) techniques tend to result in inaccuracies because of the specific characteristics of this method [1]. To estimate the response of biological tissues to the deposited energy, the absorbed dose must be accurately estimated [2].

Personalized dosimetry is a main concern when planning BT procedures [3]. In recent years interest has grown in using advanced techniques for better personalization [4,5]. Additive manufacturing as well as the virtual reality procedure have been utilized [6,7]. These techniques tend to optimize the dose distribution, as well as improve the therapeutic result.

Currently, most BT treatment planning systems use air kerma strength of the BT seed that will be used and calculate dose distribution using the inverse square of radial distance [8,9]. The limitations that arise have been considered by Task Group 43 and its updates. AAPM Task Group (TG) 43-based [10] dosimetry improved prior dose calculation formalisms for brachytherapy treatment planning. TG-43-based dosimetry exploits source-specific data, pre-calculated in a limited geometry (homogeneous water medium) [11,12]. This way, it excludes patient-specific radiation scatter conditions, tissue heterogeneity and inter-seed scattering, and attenuation effects from the dose calculation.

Moreover, TG-186 [13] updated the guidelines of TG-43, TG-43U1 and TG-43U1S1 [14]. TG-186 uses the formalism on brachytherapy source algorithm validation but proposes modifications on dosimetric calculations for clinical cases, using model-based dose calculation algorithms (MBDCAs), to ensure practice uniformity. Utilizing new guidelines, heterogeneities are taken into account in dosimetry studies.

MC is a mathematical method that exploits random sampling, the law of large numbers and the central limit theorem, to produce data that can be analysed using statistical methods and lead to a better understanding of a high complexity system [15]. The use of MC simulations for radiation therapy dosimetric studies is analysed in dedicated textbooks [16]. MC has been established as a significant tool to improve the dosimetric accuracy for brachytherapy applications providing high-quality single or multiple source data for TG-43-based Treatment Planning Systems (TPSs) [17].

This study uses a novel approach by means of advanced anthropomorphic computational models, combined with low uncertainty MC simulations. To this aim, six commercially available brachytherapy seeds were simulated using the GATE and GGEMS MC toolkits. Our objective was to generate a seed model open database, for MC dosimetric calculations in brachytherapy applications.

## 2. Materials and Methods

### 2.1. AAPM TG-43 Formalism (Anisotropy, Radial Dose, Dose Constant)

The AAPM TG-43 dosimetric formalism suggested specific quantities for the evaluation of dosimetry in brachytherapy simulations, such as the radial dose function (RDF), g(r); the anisotropy function, F(r, θ); the air kerma strength, S_k_, measured in U, where U stands for cGy cm^2^ h^−1^; the geometry factor, G(r, θ); the dose rate constant, Λ.

**Figure 1:**
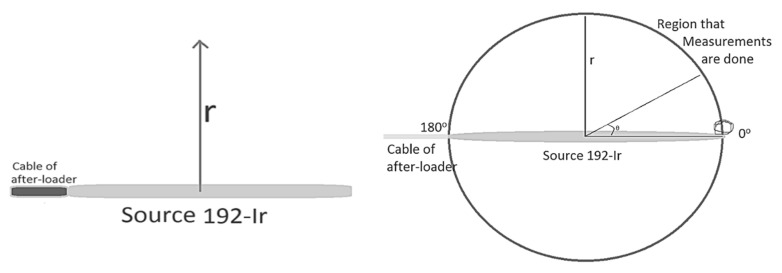
Geometric representation for the calculation of radial dose function and anisotropy function, for cylindrical sources.

Λ is defined as the dose rate per unit air kerma strength (U) at 1 cm along the transverse axis and depends on the type of source, its construction and encapsulation; G(r, θ) accounts for the distance dependence of photon fluence around a source in free space that depends on the distribution of radioactive material; F(r, θ) accounts for anisotropy of angular dose distribution due to photon absorption and scatter in the source encapsulation and in the surrounding medium; g(r) accounts for the radial dependence of dose (scatter and absorption) in the medium along the transverse axis of the source (Figure 1) [11].

The application of the TG-43 dosimetric formalism is restricted to cylindrically symmetric sources, such as the general design illustrated in Figure 1. For such sources, the dose distribution can be considered two dimensional and can be described in terms of a polar coordinate system with its origin at the source centre where ***r*** is the distance to the point of interest and ***θ*** is the angle with respect to the long axis of the source (Figure 1).

The general 2D dose rate equation from the 1995 TG-43 dosimetric formalism is given by Equation (1):

(1) $$\dot{D}=(r,\theta )=S_{K}\Lambda \frac{G_{L}(r,\theta )}{G_{L}(r_{0},\theta_{0})}g_{L}(r)F(r,\theta )$$

***r*** denotes the distance (in cm) away from the centre of the active source to the point of interest, ***r*_0_** denotes the reference distance which is specified to be 1 cm, and ***θ*** denotes the polar angle specifying the point of interest ***P*(*r*,*θ*)**, relative to the source longitudinal axis. The reference angle, ***θ*_0_**, defines the source transverse plane, and is specified to be 90° or π/2 radians (Figure 1). The Z- and Y-axis are chosen as the longitudinal and transverse axes, respectively. The origin is taken as the centre of the active part, with the positive Z-axis directed through the source tip [11].

### 2.2. Brachytherapy Seeds (Oncoseed, Isoseed, VS2000, M-19, mHDR-v1, mPDR-v2)

In this study, six brachytherapy seeds were modelled. Two Low Dose Rate (LDR) ^125^I sources, the Amersham Oncoseed 6711 and the Bebig Isoseed I25.S06; three High Dose Rate (HDR) ^192^Ir sources, Nucletron mHDR-v1 (classic), Varian VS2000 and SPEC HDR-M19; and one Pulsed Dose Rate (PDR) ^192^Ir source, the Nucletron mPDR-v2 [11].

### 2.3. MC Simulations (GATE/GGEMS)

Despite the high accuracy of MC simulations, their clinical application remains limited due to their long computational time demands. To alleviate this limitation patient-specific dose kernels can be precomputed. However, such an approach remains time consuming and demands computational resources (computer clusters), which are not easily accessible to the majority of clinical centres.

For the MC simulations in the present study GATE [18,19] and GGEMS [20] toolkits were used. GATE is a fully validated MC simulation toolkit [21,22]. The GATE version 8.2 was utilized to run simulations related to this study. A more efficient and cost-effective solution is the use of Graphical Processing Units (GPU) to accelerate MC simulations. Several GPU-accelerated MC engines, for dosimetric studies, have been proposed by different groups already [23,24,25,26]. The GGEMS toolkit [20,24] is a GPU accelerated MC dose engine based on the Geant4 physics models and has been used for dosimetry applications in prostate brachytherapy procedures [24,27,28,29]. In the present study, we evaluated the ability of GGEMS to provide accurate dosimetric data in a brachytherapy scenario using the Amersham, Oncoseed, 6711 type. Simulation results produced by both toolkits (GATE and GGEMS) were compared in terms of absorbed dose and time efficiency. It should be stated that during the validation procedure, with the TG-43, the modelling capabilities of GATE were utilized. Seed geometries were modelled using a combination of geometric volumes.

To compare these two toolkits, several simulation procedures were designed. At first, the Amersham Health 6711 brachytherapy seed was placed in the center of a sphere phantom filled with water. Then, results were compared in terms of RDF, AF and dose rate constant. In addition, dose profiles in the water sphere as well as in patient data were compared.

### 2.4. Physics List

The standard EM model of Geant4 physics list was utilized (emstandard) in both MC codes (GATE and GGEMS), incorporating all the default electromagnetic physical processes (Photoelectric, Compton, Rayleigh Scattering, Electron Ionisation, Bremsstrahlung, Electron Multiple Scattering). In such a model, electron and photon interactions are considered at energies ranging from 1 keV to 100 TeV. No energy cuts were applied in the physical processes to speed-up the simulations. Instead, the TLE actor was utilized as a variance reduction technique (VRT) for speeding up clinical simulations [30,31,32]. Furthermore, in accordance with the TG-43 protocol, the following parameters were set in GATE: Emax < 10 GeV, DEDXBinning 220 (number of bins of the DEDX table), and LambdaBinning 220 (number of bins of the mean free path table). These have been described analytically by the TG-43.

### 2.5. Phase Space Files (Blender–GATE–GGEMS)

**Figure 2:**
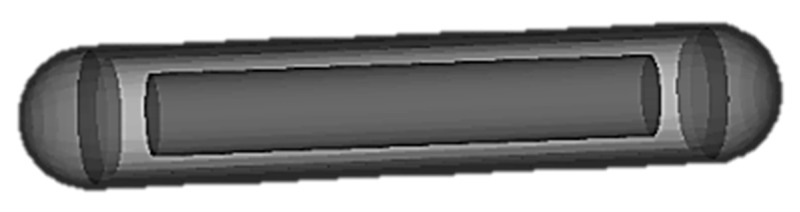
Amersham Oncoseed model in Blender.

To standardize the procedure of the present study for later use, the brachytherapy seeds were designed in the Blender [33] software as 3D triangular surface mesh models, with detailed characteristics taken from TG-43. Figure 2 represents the model of Amersham Oncoseed as it was modeled in Blender. Phase space (PHSP) files were generated for each seed in GATE using 10^6^ primaries. For this PHSP only gamma particles were recorded, and no secondary particles were taken into account.

The PHSP actor has the ability to record all the information about energy particles (type, coordinates, direction along axes, kinetic energy, weight) that enter a pre-defined volume which the actor has been attached to. The PHSP file can then provide particles’ information that has been stored, to a simulation toolkit. All PHSPs included in this study were designed to model the shape of each individual seed source. With a complicated volume, PHSP is the only way to reproduce geometry, direction and any attenuation of the radiation produced by this volume.

The PHSP is also a flexible way to reproduce any simulation using seed models without the need of modeling the initial geometry of the seed. Currently, GGEMS can use PHSPs as input, but cannot generate a PHSP file itself. Hence, GATE is used to generate the validated PHSPs of the seeds of interest and then such files are incorporated in GGEMS for performing brachytherapy simulations. Each PHSP contained 10^6^ particles.

### 2.6. Clinical Case (Brachytherapy Plan with 67 Seeds)

A clinical case of a prostate brachytherapy was simulated in both GATE and GGEMS. TG-186 guidelines were considered, to take into account the heterogeneities that are included in the models. Sixty-seven seeds were implanted in the patient’s prostate, according to the clinical treatment plan (TP) scenario. Simulations executed in GGEMS and GATE took into account tissue heterogeneities of the patient’s anatomy. The resolution of the CT data that were used is 0.78125 × 0.78125 × 2.0 mm. The actor (energy scorer) resolution was kept at the same level. To translate HU units to materials, the *setRangeToMaterialFile* was utilized. The range of HU was between −1050 and 4000. Lung, SoftTissue, ProstateICRU and bones were used as materials of the model. The material composition is the one provided by the GATE and Geant4 databases. The full geometric specification of seeds was considered during the simulations. In GATE, seeds were modelled as analytical phantoms and were attached to the voxelized phantom of the patient’s anatomy. The materials used to simulate the seeds as well as their geometry have been published in reference [12].

In GGEMS, seeds were modelled using previously generated PHSPs, as described earlier. To ensure low statistical uncertainty (<3% in the prostate), 10^8^ primaries were simulated in both GATE and GGEMS. In both cases, the Track Length Estimator (TLE) [30] was used, to reduce the statistical uncertainty of the recorded energy deposition, as well as to accelerate simulations. The TLE actor involves a local energy deposition by secondary electrons. In addition, TLE recorded the absorbed dose during the whole trajectory of each simulated energy particle, consequently reducing the required number of primaries needed to converge. Organ dose distributions were also investigated in terms of the cumulative Dose Volume Histogram (cDVH), as it is important clinical information that is utilized by any TPS during planning.

## 3. Results

### 3.1. Validation of GATE Simulated Data–TG-43 Protocol

**Figure 3:**
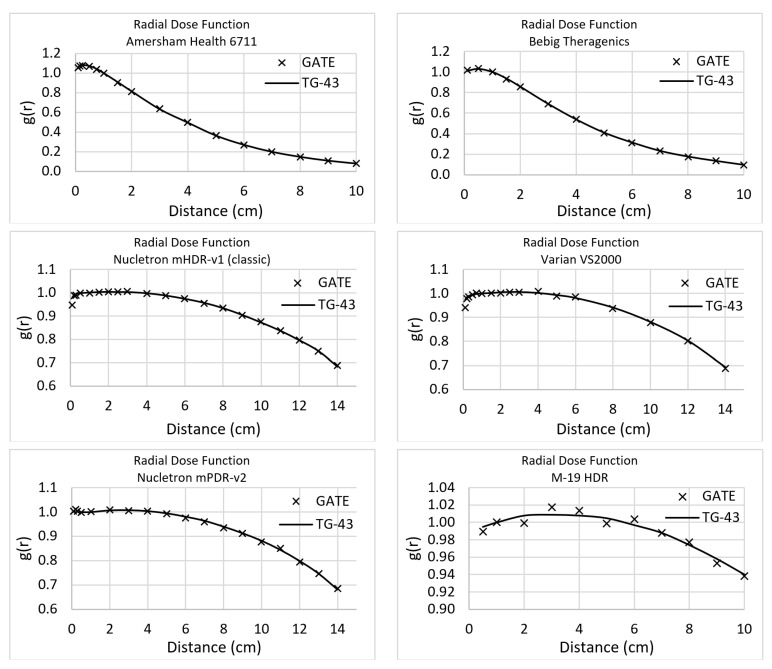
Radial Dose Function for all seeds simulated in GATE, for this study.

The comparison of the six brachytherapy seeds’ data obtained by the TG-43 with those that resulted from using MC simulations that were performed in the context of the present study are presented in this section [34]. The statistical uncertainty has been kept lower than 1% by using 10^12^ primaries. The statistical difference between the simulated and the TG-43 provided RDF values was lower than 1%, which is the limit advised for acceptance by the TG-43. Figure 3 displays the RDF comparison for every studied source. It must be mentioned that the same points were calculated for both simulation toolkits and the same points had been used by the TG-43 (even if some data are shown in continuous lines).

The anisotropy function average difference after the comparison of the simulated results with TG-43 is presented in Table 1. This study provides a database with a more analytical representation of the simulations’ outcome, as supplementary material. The statistical uncertainty was kept lower than 1% for all the cases. The average difference ranges from 1.3% to 6.0%.

Dose Rate Constant (Λ) is another variable of the TG-43 formalism used for the validation of MC calculations. The statistical difference was lower than 1% for five of the six of the seeds. The SPEC HDR-M-19 seed source provided a statistical difference up to 2.7%, which is acceptable according to TG-43 formalism (<5%). It should be stated that statistical uncertainty for this calculation is <1%. Table 2 presents the comparison of the dose rate constant for all the simulated sources, expressed in cGy h^−1^ U^−1^; U equals cGy cm^2^ h^−1^.

### 3.2. Validation of GGEMS with GATE

In this section the comparison between the validated data of GATE simulation and the data obtained with GGEMS for the Amersham Health 6711 seed is presented.

#### 3.2.1. Amersham Health 6711-Anisotropy, Radial Dose, Dose Constant, Dose Profiles

**Figure 4:**
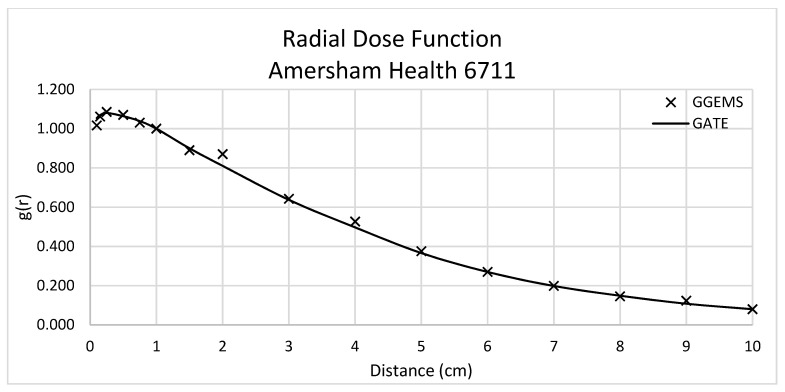
Radial Dose Function for Amersham Health 6711 (LDR). Comparison between GATE and GGEMS simulation results.

Dose rate constant, Λ, was equal to 0.923 cGy/(hU) for GGEMS, while 0.964 cGy/(hU) for GATE, with an absolute difference of 4.3%. Figure 4 depicts the comparison of radial dose function between GATE and GGEMS. The average difference of these two calculations is 1.56%, ranging between 0 and 6%.

The average difference for the anisotropy function was 9.9%, 9.8%, 5.8%, 6.1% and 7.8% for 0.5 cm, 1 cm, 2 cm, 3 cm and 5 cm distance from the source, respectively.

**Figure 5:**
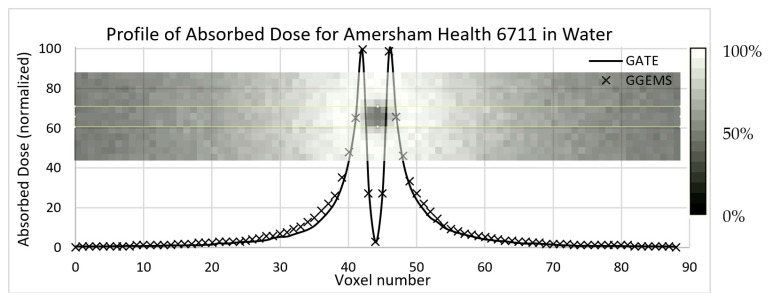
Profile of absorbed dose in water sphere (15 cm radius) of the Amersham Health 6711 Brachytherapy ^125^I seed. The voxel size is 0.25 mm, and image dimensions are 89 × 13 × 89 voxels (this does not cover the whole water sphere). The seed is in the middle and oriented towards the inside of the paper.

In Figure 5, the results of the comparison of dose profiles are shown. One seed has been placed inside a water phantom; the profile has been plotted in the central slice. Statistical uncertainty has been kept <3%, while the average difference is lower that 10% close to the source.

#### 3.2.2. One (1) Seed–Patient CT

**Figure 6:**
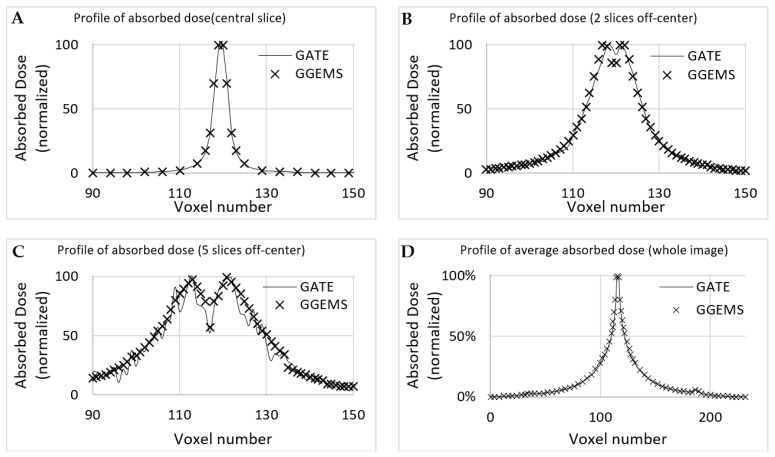
Profile of absorbed dose of the simulation of Amersham Health 6711 inserted in the CT phantom. Transverse orientation is used (as seen in Figure 7). (**A**) is the profile plot at the centre of the seed, (**B**) is the profile plot at the edge of the source, and (**C**) is a profile plot away from the seed. In (**D**), the profile plot when all slices were summed into one is depicted for the whole developed phantom.

Dose profiles were plotted for comparing both simulation codes in various CT axial images (Figure 6), as described below. The first case (A) is the profile plot at the centre of the seed, (B) is the profile plot at the edge of the source, and (C) is a profile plot away from the seed. In D, the profile plot when all slices were summed into one is depicted for the whole developed phantom. It is observed that, when calculating the absorbed dose far from the source, uncertainty is increased for GATE, while GGEMS stays stable.

Statistical uncertainty is low (<3%) close to the source, while it rises up to 15% for slices and voxels away from the source (>10 cm), due to low dose deposition. The average difference for voxels close to the source, where it only matters for these low dose rate sources, is <10%.

#### 3.2.3. Sixty-Seven (67) Seeds–CT Phantom–Clinical Case

**Figure 7:**
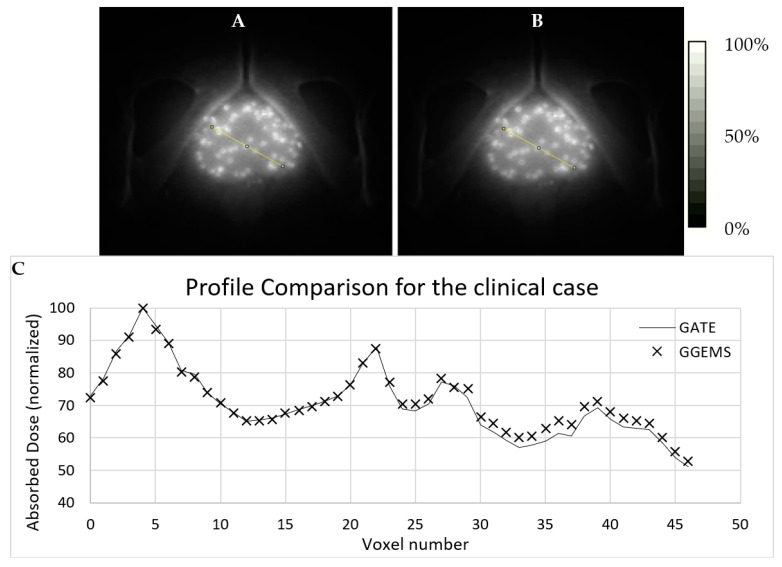
Simulation of a clinical case with 67 Amersham Health 6711 seeds in a prostate. Dose maps are shown for both toolkits (**A**) GATE and (**B**) GGEMS. White stands for maximum absorbed dose (100%). In (**C**) a profile comparison is depicted for both simulation tools. The line seen on (**A**) and (**B**) images is the region where the dose profile is taken.

**Figure 8:**
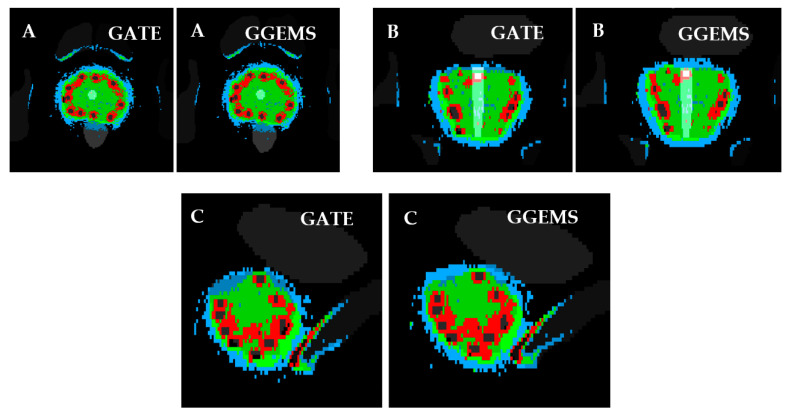
Slice of prostate irradiation, with the segmentation of organs and the deposited energy. Left: GATE simulation. Right: GGEMS simulation. (**A**) Transverse, (**B**) Coronal and (**C**) Sagittal slice. With colour, areas with similar dose are shown. Blue: Dose > 50%. Green: Dose > 100%. Red: Dose > 150%.

For the brachytherapy case under investigation, the generated dose maps with GATE and GGEMS were compared in terms of cDVH metrics and the absorbed dose distribution. No significant statistical difference between the dose maps of the two dose calculation engines was found in the qualitative inspection of the dose maps (Figure 7 and Figure 8).

Figure 7 and Figure 8 present the comparison of the dose maps generated by GATE and GGEMS. The average difference on the profiles (Figure 7) was 2.1% and the maximum statistical uncertainty was lower than 6.2%.

**Figure 9:**
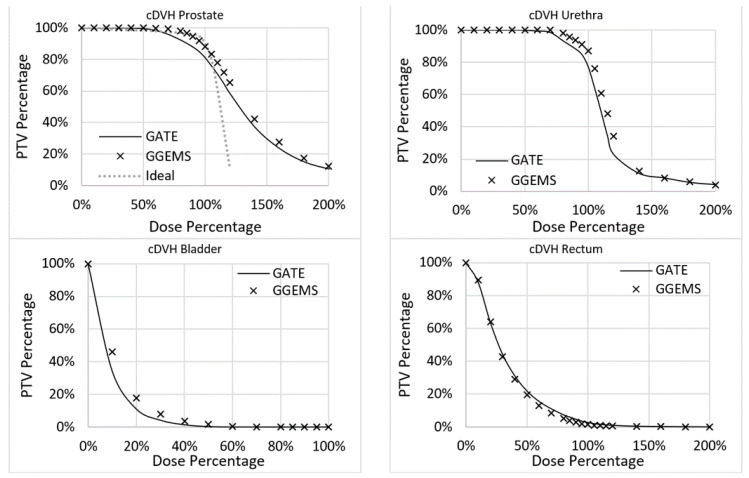
cDVH for the four segmented organs.

Furthermore, variations in the DVH histograms were found (Figure 9). More specifically, for the prostate, the average difference between the two codes was 3.4%, while the maximum was 7.3%. For the urethra, the average difference between the two codes was 3.5%, while the maximum was 13.3%. For the rectum, the average difference between the two codes was 1.1%, while the maximum was 2.6%. For the bladder, the average difference between the two codes was 1.7%, while the maximum was 11.9%. No real data of a TPS existed to compare the two codes with clinical data.

It must be stated that GATE is implemented only in CPUs, while GGEMS can be implemented in GPUs. This means that they cannot be directly compared as they are executed in different processors. Nevertheless, significant difference between GGEMS and GATE was found in the computational time required for the two simulations. The required time for the execution of the GATE clinical simulation was 1 h for 10^8^ primaries in a computer cluster of 120 processors (ARIS High Performance Computers). The same simulation was executed in GGEMS in only 162 s on a single NVIDIA 960M GTX GPU, on a single PC. The presented significant time reduction of MC simulation using GGEMS while keeping the same computational accuracy, compared to GATE, may allow the integration of the proposed method in routine clinical practice.

## 4. Discussion

This study showed that GATE is a well validated tool and could simulate with the necessary accuracy brachytherapy seeds. Low uncertainty results were produced for the characteristics of the six brachytherapy seeds that were modelled and simulated. Radial dose function, anisotropy function and the dose rate constant were calculated and validated the simulation toolkits that were utilized in the context of this study.

In terms of statistical differences, it must be stated that experimental procedures used in clinical practice (in vivo) contain geometrical limitations when compared to the simulation outcome. For example, during the experimental procedure, dosimeters can be a limited number and can only be placed in specific positions. During the simulation, such a consideration does not exist. That could be a reason why differences up to 6% were observed in some AF results.

Moreover, the required time for the execution of the GATE clinical simulation was 1 h for 10^8^ primaries in a computer cluster of 120 processors (ARIS Hyper computer). To achieve lower statistical uncertainty (<1%) for the whole body 10^10^ primaries could be used. This would result to an increase in computational time by at least 100 times. Thus, we did not produce such results.

This study was also extended in using GGEMS to run the same calculations. The purpose was to investigate the probability of using such a toolkit during the clinical procedure, in real time. GGEMS was validated, by comparing its results to the same simulations run on GATE. For this investigation, a prostate clinical case was modelled and simulated in both GATE and GGEMS. The results showed that both tools produced results of comparable accuracy. GGEMS accelerated the procedure more than twenty times.

The implementation of personalized dosimetry considering tissue heterogeneity in clinical practice will remain a hot topic of research. This study tried to add some knowledge concerning this endeavour by exploiting GPU capabilities. GGEMS was able to produce the same results as GATE, which has already been validated in the field of radiation dosimetry. The relative average difference of GATE with GGEMS was lower than 4% for the investigated case (Figure 9). This difference is attributed to implementation differences between the two toolkits. It was not related to the physics used, since both toolkits are based on the same Geant4 physics module, and use the same physics list, namely standard (default).

GATE can be accelerated with the use of computer clusters. This is a high-cost solution and the access to such a cluster may be restricted for a standard clinical centre. GGEMS is a more user-friendly platform, which can produce fast and reliable outcomes for specific cases (e.g., brachytherapy cases using the available seed models, and patient-specific computational phantoms). Hence, the computational time of MC dosimetry may be reduced from lots of hours to a few minutes, as presented in the current study.

Furthermore, this study presented tools that were produced for the evaluation of various simulation outcomes. The user can evaluate the treatment plan of a brachytherapy case, by considering isodose curves and organ-specific cDVHs, including tissue heterogeneities in the voxel level. Improved segmentation techniques may allow producing computational phantoms of improved quality that may lead to more accurate simulations, which may be a future study of our group.

## 5. Conclusions

In this study a dataset of PHSPs for six commonly used commercial brachytherapy seeds was generated with the GATE MC toolkit. The dosimetry was fully validated according to the TG-43 formalism. In addition, a rather new GPU MC toolkit, GGEMS, was evaluated, incorporating the generated PHSPs for a clinical application. Both GATE and GGEMS provided an accurate dosimetry assessment, with the difference in the computational time. GGEMS seems to be an alternative option for fast brachytherapy calculation in realistic clinical scenarios, while GATE is an accurate tool but rather slow for such applications. Based on the fact that the average differences overall were fairly low, GGEMS could also operate as a quick validation for complex procedures.

More research is deemed necessary concerning the implementation of personalized dosimetry in clinical conditions. Nevertheless, this study made a step forward in this direction. A methodology to perform MC simulations using GGEMS and clinical CT images with several realistic models of brachytherapy seeds, in approximately five (5) minutes, was reported in this article. The outcome of this method was evaluated by means of several tools used in clinical practice (i.e., cDVH graphs, isodose curves, etc.)

## Figures and Tables

**Table 1:** Average Statistical Difference of Anisotropy Function, for all seeds, when simulation output is compared with TG-43 data.

Source	Distance (cm)	Average Difference (%)
Amersham Health 6711 ^125^I	0.5	2.8
	1.0	3.0
	2.0	2.8
	3.0	2.6
	5.0	3.8
Bebig Theragenics ^125^I	0.5	6.0
	1.0	3.7
	2.0	3.8
	3.0	3.1
	5.0	5.4
Nucletron mHDR-v1 ^192^Ir	0.5	3.5
	1.0	4.1
	2.0	3.5
	3.0	4.1
	5.0	4.5
Varian VS2000 ^192^Ir	0.5	2.6
	1.0	1.4
	2.0	-
	3.0	2.8
	5.0	2.1
Nucletron mPDR-v2 ^192^Ir	0.5	3.4
	1.0	2.9
	2.0	1.6
	3.0	1.7
	5.0	2.0
SPEC Μ-19 ^192^Ir	0.5	3.4
	1.0	2.7
	2.0	3.7
	3.0	3.7
	5.0	3.8

**Table 2:** Dose Rate Constant (in cGy h-1 U-1), for all seeds, when simulation output is compared with TG-43 data.

Source	GATE	TG-43	Difference %
Amersham Health 6711 ^125^I	1.012	1.012	0.0
Bebig Theragenics ^125^I	1.012	1.012	0.0
Nucletron mHDR-v1 ^192^Ir	1.114	1.109	0.5
Varian VS2000 ^192^Ir	1.097	1.098	0.1
Nucletron mPDR-v2 ^192^Ir	1.101	1.108	0.6
SPEC Μ-19 ^192^Ir	1.1	1.13	2.7

## Data Availability

The data that support the findings of the present study are available from the corresponding author upon reasonable request.
